# Lessons learned during implementation of MR-guided High-Intensity Focused Ultrasound treatment of uterine fibroids

**DOI:** 10.1186/s13244-021-01128-w

**Published:** 2021-12-18

**Authors:** K. J. Anneveldt, I. M. Verpalen, I. M. Nijholt, J. R. Dijkstra, R. D. van den Hoed, M. van’t Veer-ten Kate, E. de Boer, J. A. C. van Osch, E. Heijman, H. R. Naber, E. Ista, A. Franx, S. Veersema, J. A. F. Huirne, J. M. Schutte, M. F. Boomsma

**Affiliations:** 1 Department of Radiology, Isala Hospital, Zwolle, The Netherlands; 2 Department of Gynecology, Isala Hospital, Zwolle, The Netherlands; 3 Department of Radiology, Amsterdam University Medical Center, location AMC, Amsterdam, The Netherlands; 4 Department of Medical Physics, Isala Hospital, Zwolle, The Netherlands; 5 Department of Diagnostic and Interventional Radiology, University of Cologne, Cologne, Germany; 6 Department of Oncology, Philips Research Eindhoven, Eindhoven, The Netherlands; 7 Department of Anesthesiology, Isala Hospital, Zwolle, The Netherlands; 8 Department of Internal Medicine, Section of Nursing Science, Erasmus Medical Center, Rotterdam, The Netherlands; 9 Department of Obstetrics and Gynecology, Erasmus Medical Center, Rotterdam, The Netherlands; 10 Department of Reproductive Medicine and Gynecology, University Medical Center Utrecht, Utrecht, the Netherlands; 11 Department of Gynecology, Amsterdam University Medical Center, location AMC, Amsterdam, The Netherlands

**Keywords:** Magnetic resonance imaging, Minimally invasive surgery, Uterine fibroids, Learning-curve, MR-HIFU

## Abstract

**Background:**

Although promising results have been reported for Magnetic Resonance image-guided High-Intensity Focused Ultrasound (MR-HIFU) treatment of uterine fibroids, this treatment is not yet widely implemented in clinical practice. During the implementation of a new technology, lessons are learned and an institutional learning-curve often has to be completed. The primary aim of our prospective cohort study was to characterize our learning-curve based on our clinical outcomes. Secondary aims included identifying our lessons learned during implementation of MR-HIFU on a technical, patient selection, patient counseling, medical specialists and organizational level.

**Results:**

Our first seventy patients showed significant symptom reduction and improvement of quality of life at 3, 6 and 12 months after MR-HIFU treatment compared to baseline. After the first 25 cases, a clear plateau phase was reached in terms of failed treatments. The median non-perfused volume percentage of these first 25 treatments was 44.6% (range: 0–99.7), compared to a median of 74.7% (range: 0–120.6) for the subsequent treatments.

**Conclusions:**

Our findings describe the learning-curve during the implementation of MR-HIFU and include straightforward suggestions to shorten learning-curves for future users. Moreover, the lessons we learned on technique, patient selection, patient counseling, medical specialists and organization, together with the provided supplements, may be of benefit to other institutions aiming to implement MR-HIFU treatment of uterine fibroids.

*Trial registration* ISRCTN14634593. Registered January 12, 2021—Retrospectively registered, https://www.isrctn.com/ISRCTN14634593.

**Supplementary Information:**

The online version contains supplementary material available at 10.1186/s13244-021-01128-w.

## Key points

- Our learning-curve flattened after 25 uterine fibroid MR-HIFU treatments.
- A completed institutional learning-curve is a requirement for MR-HIFU adoption.
- Implementation barriers include technique, patient selection, patient counseling, specialists and organization.
- Early involvement of all relevant parties is essential for adequate implementation.

## Background

Uterine fibroids are benign tumors and clinically apparent in 25% of women during their reproductive age. They cause symptoms such as heavy menstrual bleeding, abdominal pain or pressure and subfertility [1]. In recent years, new (innovative) therapies for the treatment of uterine fibroids became available. These include hormonal medical therapy such as GnRH analogs or oral contraceptives and non-hormonal medical therapy such as tranexamic acid or non-steroidal anti-inflammatory drugs. Less invasive, non-medical treatment options consist of hysteroscopic removal of submucosal fibroids, endometrial ablation and uterine artery embolization [2]. However, despite promising clinical outcomes, implementation in daily practice is challenging [3]. One non-invasive treatment that has become available is Magnetic Resonance image-guided High-Intensity Focused Ultrasound (MR-HIFU). This technique can be used for a wide range of benign and malignant diseases including uterine fibroids [4]. Complication rate is low and recovery fast [5]. Furthermore, because of the uterus-saving character of this treatment, women with a wish to conceive can be treated by MR-HIFU [6]. Therapeutic success of MR-HIFU is often measured by the percentage of non-perfused volume (NPV%) compared to the total volume of the fibroid pre-treatment. A high NPV% is closely related to treatment results and, in particular, clinical effectiveness [5]. To achieve a high NPV%, proper screening of eligible patients is essential but remains a challenging task that needs to be further improved [7].

Clinical and technical aspects of the MR-HIFU treatment have already been reported in detail [5, 8, 9]. Learning-curves of the MR-HIFU treatment for fibroids and suggestions on how to implement MR-HIFU treatment have also been described , however, were not the primary goal of these studies [8, 10, 11]. In the present study, we assessed the learning-curve during the implementation of the MR-HIFU treatment of uterine fibroids in our center as our primary objective. Our secondary objective was to inventory all hurdles we needed to overcome and the lessons we learned during the implementation process. In this way, we aimed to facilitate future implementation of this new innovative technique.

## Methods

### Study design and protocol

We designed a single-arm prospective cohort study (the Myoma Screening Study; MaSS; registry ID ISRCTN14634593). This study consisted of two parts; both parts were approved by our medical ethical board and participants needed to sign informed consent before participating. The implementation of the MR-HIFU treatment described in this article concerns participants of the second part, the MaSSII study.

### MaSSI

In the first part (MaSSI; protocol ID NL53499.075.15) we aimed to get an overview of the uterine fibroid tissue type distribution using multiparametric magnetic resonance imaging (MRI) parameters (sagittal and axial T2-weighted turbo spin echo, T1-weighted contrast-enhanced 3D fast field echo, short-TE and long-TE DWI series and T2-mapping, Additional file 1) on a 1.5-T Achieva MRI scanner (Philips Healthcare, Best, The Netherlands) [7]. All women consecutively visiting our gynecology department between December 2015 and January 2019 because of symptomatic uterine fibroids, as confirmed by vaginal ultrasound, were offered an MRI scan after counseling and signing informed consent, independent of their eligibility for the MR-HIFU treatment.

### MaSSII

**Fig. 1:**
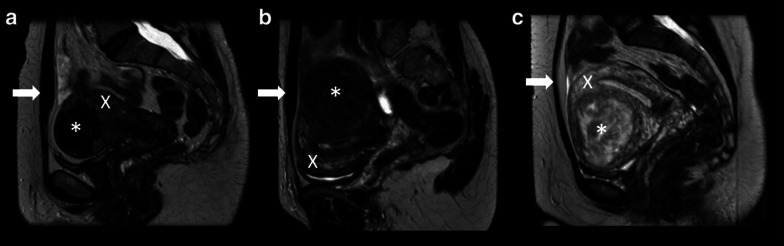
Funaki classification. **a** Funaki I fibroid (signal intensity lower than myometrium and muscle); **b** Funaki II fibroid (signal intensity lower than myometrium, but higher than muscle); **c** Funaki III fibroid (signal intensity higher than muscle and myometrium). Asterisks (*) are located in the uterine fibroid. Arrows point at abdominal muscle. Cross (X) is located in myometrium tissue

The primary aim of the second part (MaSSII; protocol ID NL56182.075.16) was to explore whether biomarkers found by the multiparametric MaSSI MRI scan could predict MR-HIFU treatment outcome. Women participating in the MaSSI study between June 2016 and January 2019 and eligible for MR-HIFU, were offered the MR-HIFU treatment option. Inclusion criteria were women with uterine fibroid-related symptoms, aged between 18 and 59 years and pre- or perimenopausal status. Women were excluded when post-menopausal, pregnant, not willing or able to sign informed consent, had a wish to conceive, a BMI > 40 kg/m^2^, had a previous embolization or contra-indications to undergo an MRI scan. Based on the MaSSI MRI scan women were eligible in case of a subcutaneous fat layer < 4 cm, a fibroid diameter between 1 and 10 cm, one or two dominant fibroid(s) likely to cause the clinical symptoms and no calcified fibroids. The Funaki classification, which classifies fibroids into Funaki type 1, 2 or 3 fibroids based on signal intensity on T2-weighted MRI images, was also used as a screening tool (Fig. 1) [7, 12]. Fibroids classified as a Funaki type 1 or 2 fibroid were considered eligible for the MR-HIFU treatment. Relative contra-indications of MR-HIFU included Funaki type 3 fibroids, interposed bowel loops or ovaries and a retroverted uterus. MaSSI participants who also participated in the MaSSII study were again counseled and signed a second informed consent form. After signing this informed consent, women were asked to fill out the Uterine Fibroid Symptom and Health-Related Quality of Life questionnaire (UFS-QoL) [13].

### MR-HIFU treatment

#### Pre-treatment

When women were considered eligible for MR-HIFU treatment and willing to participate, they were screened at the anesthesiology department and received information about the conscious sedation during the MR-HIFU procedure. The evening before the treatment, women had to shave their lower abdomen and had to fast overnight. In the morning at admission to day care, women received a bladder catheter, an enema, an intravenous line and pre-medication (Additional file 1). Women were placed in prone position in the MRI scanner, and a pre-treatment MRI scan was performed for a final fibroid position check. The MR-HIFU treatment was performed on the Sonalleve V1 (Profound Medical Inc., Mississauga, Canada), integrated into a 1.5-T Achieva MRI scanner (Philips Healthcare, Best, The Netherlands). Our sedation protocol included continuous propofol 20 mg/ml infusion between a 1 ml/hour and 12 ml/hour rate and administration of fentanyl bolus of 25 µg/0.5 ml or 50 µg/1.0 ml (Additional file 1) depending on experienced pain.

#### Treatment

MR-HIFU fibroid ablation combines high-intensity focused ultrasound with real-time MRI. A focused ultrasound beam targets uterine fibroid tissue and induces coagulative necrosis [14]. Safety is provided by MR thermometry that measures almost real-time heating of the targeted tissue and critical surrounding structures [15]. We aimed for complete ablation of the fibroid [16], and our therapy strategy was to ablate the complete posterior part of the fibroid first, followed by the middle and anterior part of the fibroid [17].

During the treatment, both the patient and the attending radiologist could press an emergency button if necessary, which would result in an immediate stop of the current sonication. After the last sonication, a contrast agent (gadoteric acid—gadoterate meglumine, 0.1 mmol/kg) was administered to assess the NPV%.

#### Post-treatment

After the procedure, patients stayed at day care for a few hours. Before discharge, the radiologist visited the patient to check for vital parameters, adverse events like (radiating) pain and possible signs of skin burn of the abdomen. If no irregularities were found, patients could leave the hospital the same day. Follow-up by the gynecologist was planned one week after treatment, and 3 and 6 months post-treatment at the outpatient clinic (Additional file 1). Recovery, possible adverse events and the decrease in symptoms were discussed with the patient during these follow-up appointments.

### Data collection

Data concerning the treatment (e.g., treatment time, reached NPV%) were collected in a standardized form, and an MRI report was added to the electronic patient file (Additional file 1). In case more than one fibroid was treated, NPV% of all fibroids was collected. When complete ablation was not achieved, the most likely reason for this was recorded after internal discussion within the treatment team. Six months after treatment, a follow-up MRI scan was performed to measure the fibroid's size and the remaining NPV%. Fibroid volume and NPV%, collected from the screening MRI scan, the MRI scan immediately after treatment and the 6-month follow-up MRI scan, were measured by K.A. and I.V., both two years’ of experience with uterine fibroid MR-HIFU, using IntelliSpace Portal (ISP) software (Philips Healthcare) by semiautomatic segmentation in the tumor tracking function with review and manual correction of the segmentation [7]. In case more than one fibroid was treated, volume (changes) and NPV% (changes) were measured for all treated fibroids. Adverse events during recovery were recorded in the electronic patient file and classified according to the classification of surgical complications, ranging between grade I (any deviation from the normal postoperative course) and grade V (death of a patient) [18].

Patients received the UFS-QoL questionnaire at 3-, 6- and 12-month follow-up. Differences by 10 points between baseline and follow-up on the 0–100 scale were considered clinically relevant [5].

In February 2020, after an MaSSII protocol addendum was approved (dated 07-11-2019) by our medical ethical board, manually electronic patient file search was performed to screen for possible reinterventions and pregnancy outcomes of all included patients. Second MR-HIFU treatments for different fibroids were not considered as reinterventions, nor were (re)start of medication or an intra-uterine device. Women were requested not to fill out the follow-up UFS-QoL questionnaire after a reintervention.

### Assessment of the learning-curve

During MaSSII inclusions, all failed treatments were logged and analyses to determine the most probable cause of failure took place immediately by the involved treatment team. When solutions were available, they were directly implemented in upcoming treatments and date of implementation was recorded. After finishing MaSSII inclusions, all causes of treatment failure were categorized and their occurrence in time, revealed an expected learning-curve per type of failure. We then evaluated our expected learning-curve by comparing reached NPV%, symptom and QoL improvement and reintervention rates between those women treated during and those women treated after completing our learning-curve.

### Evaluation of implementation process

While implementing MR-HIFU in our hospital, different process steps were taken (Table 1) [19]. The implementation process was coordinated by an MR-HIFU radiologist who was appointed as principal investigator. A dedicated multidisciplinary team was installed, including two gynecologists, four (intervention) radiologists, a medical clinical physicist and an anesthesiologist. This multidisciplinary team defined the implementation goals together with additional stakeholders in our institution after an inventory of current uterine fibroid healthcare in our hospital was executed. Stakeholders included other physicians, hospital board members, administration staff, financial experts, epidemiologists, technicians and nursing staff of the gynecology, radiology and anesthesiology department. A PhD candidate was responsible for the documentation of all steps in the implementation process, and communication between gynecology, radiology and anesthesiology departments. When facing implementation hurdles or treatment failures, consultation between members of the study team led to the development and selection of improvement suggestions. The formulated lessons learned as a result of these consultations, were categorized on a technical, patient screening, patient counseling, medical specialist or organizational level. Alterations on these levels were documented in the relevant protocols, presented to the involved parties and carried through during the MaSSII study.

**Table 1:** Different steps used during implementation process

	Description of step
Step 1	Development proposal of change
Step 2	Analysis of actual care, defining implementation goals
Step 3	Problem analysis, target group and setting
Step 4	Development and selection of interventions
Step 5	Develop, test and execute implementation plan
Step 6	Integration into daily practice
Step 7	Evaluation: reflection on outcome measures

### Statistics

Statistical analyses were performed using IBM SPSS version 26. Categorical data were presented as numbers and percentages. Continuous variables were presented as mean (SD ±) in case of normal distribution or median (range) in case of skewed distribution. Distribution was assessed by the Shapiro–Wilk test and complemented by plots.

Differences between symptom severity (transformed Symptom Severity Score; tSSS), QoL (transformed Health-Related Quality of Life score; tHRQL), NPV% and fibroid volume at baseline/directly post-treatment and follow-up were tested by means of the Wilcoxon signed rank test. Differences between age, BMI, fibroid diameter, tSSS, tHRQL, NPV% and fibroid volume of women that were treated during the learning-curve phase and women that were treated after this phase were assessed by the Mann–Whitney *U* test. A *p* value of < 0.05 was considered significant. Multiple testing correction was performed using the Holm–Bonferroni method. To estimate confounding by loss-to-follow-up, we compared NPV% between patients who completed the questionnaires during follow-up and patients who were lost to follow-up.

## Results

In total 168 women were included in the MaSSI study. Seventy women (41.7%) with 102 fibroids were treated as part of the MaSSII study. Demographic characteristics and fibroid characteristics are described in Table 2. Four of seventy women were treated twice as a result of a failed procedure at the first attempt, and two other women were treated twice for different fibroids. Thus, a total of 76 treatments (64 women with one treatment and six women with two treatments) could be analyzed in more detail.

**Table 2:** Demographic and clinical characteristics of 70 women who underwent MR-HIFU treatments

Characteristics of all 70 women		Characteristics of first 22 women		Characteristics of remaining 48 women	
Age (years) median [range] 46.0 [26–57]		Age (years) median [range] 47.0 [41–57]		Age (years) median [range] 45.0 [26–54]*	
BMI (kg/m^2^) median [range] 24.3 [17.5–35.5]		BMI (kg/m^2^) median [range] 24.0 [20.4–31.9]		BMI (kg/m^2^) median [range] 24.6 [17.5–35.5]	

Characteristics of all 76 treatments		Characteristics of first 25 treatments		Characteristics of remaining 51 treatments	
Amount of fibroids treated *N* (%)		Amount of fibroids treated *N* (%)		Amount of fibroids treated *N* (%)	
1	61 (80.3%)	1	24 (96.0%)	1	37 (72.5%)
2	6 (7.9%)	2	1 (4.0%)	2	5 (9.8%)
3	6 (7.9%)	3	0	3	6 (11.8%)
4	2 (2.6%)	4	0	4	2 (3.9%)
5	1 (1.3%)	5	0	5	1 (2.0%)

Characteristics of all 102 fibroids		Characteristics of first 21 fibroids		Characteristics of remaining 81 fibroids	
Location	*N* (%)	Location	*N* (%)	Location	*N* (%)
*Submucosal*	27 (26.5%)	*Submucosal*	6 (28.6%)	*Submucosal*	21 (25.9%)
*Intramural*	26 (25.5%)	*Intramural*	3 (14.3%)	*Intramural*	23 (28.4%)
*Subserosal*	33 (32.4%)	*Subserosal*	9 (42.9%)	*Subserosal*	24 (29.6%)
*Hybrid*	16 (15.7%)	*Hybrid*	3 (14.3%)	*Hybrid*	13 (16.0%)
Funaki type	N (%)	Funaki type	N (%)	Funaki type	N (%)
*1*	10 (9.8%)	*1*	1 (4.8%)	*1*	9 (11.1%)
*2*	83 (81.4%)	*2*	19 (90.5%)	*2*	64 (79.0%)
*3*	9 (8.8%)	*3*	1 (4.8%)	*3*	8 (9.9%)
Diameter (cm) median [range] 4.8 [1.4–18.1]		Diameter (cm) median [range] 6.0 [1.7–10.3]		Diameter (cm) median [range] 4.6 [1.4–18.1]	

The 22 women treated in the first 25 treatments (third column) were compared to the 48 women treated in the remaining 51 MR-HIFU treatments (fifth column)

*p =  < 0.05 between the first 22 and second 48 women treated

The median of the NPV% after treatment was 66.5% (range: 0–120.6; Table 3). In seventeen out of seventy women, a grade 1 adverse event was reported on treatment day [18]. This included mostly pain or nausea. One woman experienced strength loss in one leg, which was self-limiting. Another woman had a third-degree skin burn (grade 3b adverse event) which needed additional recovery surgery. During follow-up, one woman suffered from pain in her lower arm as a result of nerve compression due to the prone position during MR-HIFU, which resolved without sequelae. Two women experienced a urinary tract infection and were treated with antibiotics. At 12 months, 36 patients were lost to follow-up for the UFS-QoL questionnaires since they did not fill in the questionnaire, even after being reminded both digitally and by phone, or they had undergone a reintervention. There was no statistically significant difference in NPV% between women who completed all questionnaires and those who did not (*p* = 0.13). tSSS was significantly reduced at all follow-up points when compared to baseline, tHRQL score was significantly increased compared to baseline (Table 4). The clinically relevant 10-point difference was reached as well.

**Table 3:** Treatment results and follow-up data

Treatment outcomes	*N* (%), median [range]
NPV% directly after treatment	66.5% [0–120.6]
First 25 treatments: 21 fibroids	44.6% [0–99.7]*
Remaining treatments: 81 fibroids	74.7% [0–120.6]**
Adverse events per woman	
Grade 1 adverse event on treatment day	17/70 (24.2%) pain/nausea
Grade 3b adverse event on treatment day	1/70 (1.4%) 3th degree skin burn
Grade 1 adverse event follow-up	22/70 (31.4%) pain/ bleeding
Grade 2 adverse event follow-up	2/70 (2.9%) urinary tract infection
Adverse events needing treatment	3/70 (4.3%) antibiotics/operation
Volume decrease in fibroids with an available MRI scan at 6-month follow-up	42.4% [− 173.2 to 100]
First 25 treatments: 14 fibroids	31.7% [7.1–62.2]
Remaining treatments: 70 fibroids	48.3% [− 173.2 to 100]
Reintervention rate per woman	19/70 (27.1%)
Hysterectomy	10/19 (52.6%)
Myosure	2/19 (10.5%)
UAE	4/19 (21.1%)
MR-HIFU	4/19 (21.1%)
First 22 women	10/22 (45.5%)
Remaining 48 women	9/48 (18.8%)
Moment of reintervention	8 months [1–27]
Follow-up duration	24 months [14–44]
Failure of treatment	19/76 (25.0%)
First 25 treatments	12/25 (48.0%)
Remaining 48 treatments	7/51 (13.7%)
Kind of failures	
Treatment	13/19 (68.4%)
Heating	6/19 (31.6%)

*p =  < 0.05 between the first 25 and remaining 51 treatments

**An NPV% of > 100% could be found when the measured NPV volume exceeded the measured volume of the fibroid at screening MRI scan. This could be caused by either fibroid growth, measurement accuracies or increase in the fibroid directly after treatment due to treatment effect

**Table 4:** UFS-QoL questionnaire scores at baseline, 3-, 6- and 12-month follow-up

All women	Baseline *n* = 70	3 m * n* = 61	6 m * n* = 55	12 m * n* = 37
tSSS	50.4 ± SD15.9	36.0 ± SD16.9 Δ-14.4*	31.2 [range: 0–78.1] Δ-19.2*	32.6 ± SD18.1 Δ-17.8*
tHRQL	57.4 ± SD19.0	70.0 [range: 13–100] Δ12.6*	80.0 [range: 10–100] Δ22.6*	73.5 ± SD19.3 Δ16.1*

First 22 women	Baseline *n *= 22	3 m * n* = 17	6 m * n* = 17	12 m * n* = 13
tSSS	48.6 ± SD14.8	33.6 ± SD15.3 Δ-15*	32.0 ± SD23.9 Δ-16.6*	30.5 ± SD18.1 Δ-18.1*
tHRQL	63.5 ± SD17.9	72.5 ± SD15.3 Δ9	83.0 [range: 10–98] Δ19.5	78.0 ± SD17.0 Δ14.5*

Remaining 48 women	Baseline *n *= 48	3 m * n* = 44	6 m * n* = 38	12 m * n* = 24
tSSS	51.3 ± 1SD6.5	37.0 ± SD17.5 Δ-14.3*	33.5 ± SD18.9 Δ-17.8*	33.8 ± SD18.4 Δ-17.5*
tHRQL	54.6 ± SD19.0	70.1 ± SD19.5 Δ15.5*	79.5 [range: 21–100] Δ24.9*	71.0 ± SD20.4 Δ16.4*
*p* value	*p* = 0.33 and * p* = 0.06	*p* = 0.56 and * p* = 0.78	*p* = 0.69 and * p* = 1.00	*p* = 0.53 and * p* = 0.37

Showing all women and divided in the first 22 and second 48 women treated. Δ shows the absolute difference with baseline, and *shows a significant difference (*p* =  < 0.05) compared to baseline. The bottom row of the table shows *p* values of the difference between the first 22 and second 48 women treated for tSSS or tHRQL at that particular time point, tested by Mann–Whitney *U* test. Multiple testing correction was performed using the Holm–Bonferroni method

Median follow-up time for the assessment of reinterventions was 24 months (range: 14–44). A total of nineteen women (27.1%) needed a reintervention. One woman needed two reinterventions. The median NPV% post-treatment of women that underwent a reintervention was 4.5% (range: 0–98.3). Fifteen of twenty reinterventions (75%) took place in the first 12 months and nineteen of twenty reinterventions (95%) in the first 24 months after the initial MR-HIFU treatment.

### Learning-curve

A total of 25% (19/76) of the treatments could be classified as treatment failures due to different reasons (Table 5).

**Table 5:** Overview of failed treatments, reason of failure and possible solution

Treatment number	Patient number	Category failure	Kind of failure	Possible solution
1	1	Treatment	Interposition of bowel	New manipulation protocol
2	2	Treatment	Abdominal scar in pathway	New manipulation protocol
3	2	Treatment	Abdominal scar in pathway	New manipulation protocol
4	3	Treatment	Interposition of bowel	New manipulation protocol
5	3	Treatment	Interposition of bowel	New manipulation protocol
6	4	Treatment	Interposition of bowel	New manipulation protocol
7	5	Treatment	Interposition of bowel, part unreachable because of distance and abdominal scar	New manipulation protocol
8	6	Treatment	Interposition of bowel and small fibroid	New manipulation protocol and breath hold instructions
9	7	Treatment	Pain, interposition of bowel	New manipulation protocol and alterations in sedation protocol
10	8	Heating	Pain, no adequate heating and part unreachable	Alterations in sedation protocol and adequate screening of patients
11	9	Treatment	Pain during treatment	Alterations in sedation protocol
12	10	Heating	No adequate heating	Adequate screening of patients
13	11	Heating	No adequate heating	Adequate screening of patients
14	12	Heating	No adequate heating	Adequate screening of patients
15	13	Treatment	Interposition of bowel	New manipulation protocol
16	14	Heating	No adequate heating	Adequate screening of patients
17	15	Heating	No adequate heating	Adequate screening of patients
18	16	Treatment	Interposition of bowel	New manipulation protocol
19	17	Treatment	Interposition of bowel	New manipulation protocol

During thirteen treatments, bowels, ovaries or an abdominal scar obstructed the sonication beam pathway to such an extent that a too small part of the fibroid was accessible for sufficient sonications or treatments failed due to patients experiencing too much pain leading to preliminary abortion of sonications. The remaining six treatment failures were all due to inadequate heating of the fibroid tissue.

**Fig. 2:**
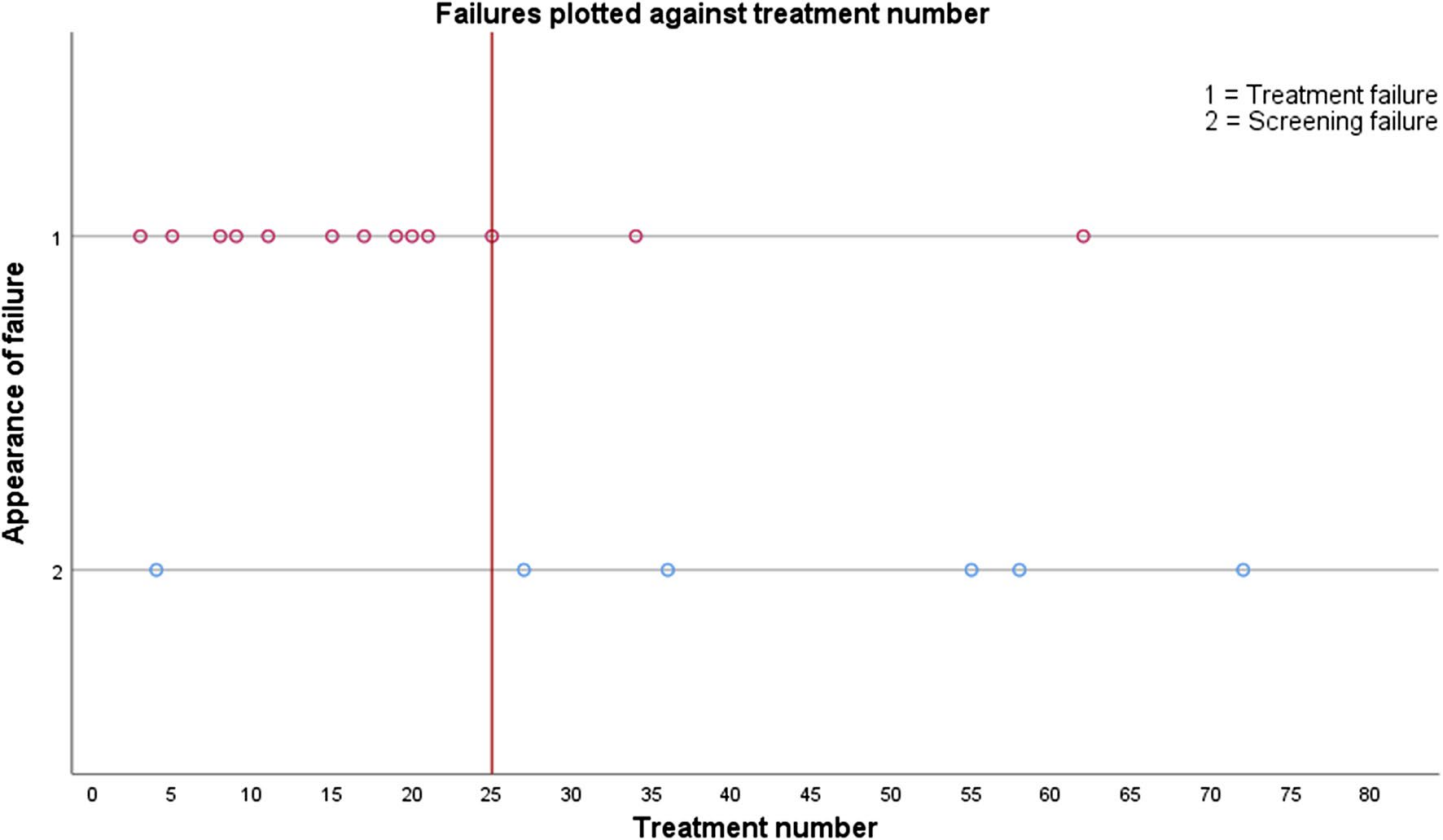
Appearance of treatment failure when plotted against the number of treatments. Blue dots represent treatment failures; pink dots represent screening failures

The occurrence in time of the nineteen treatment failures was analyzed. Of these failures, twelve occurred within the first 25 treatments, resulting in a failure rate of 48% (12/25, Fig. 2). The remaining treatment failures occurred after the 25 treatments, resulting in a failure rate of 14% (7/51). This means that not all failures occurred during our learning-curve. However, the cause of the failures during and after the learning-curve differed. Eleven of the twelve failures during first 25 treatments, could be attributed to inexperience and solved by alterations in the treatment protocol. Six out of seven failures, after completing the learning-curve, did not seem to be the result of inexperience, but rather the result of the extension of the inclusion criteria and as a result including more challenging cases with a higher risk on failure. We therefore considered the first 25 treatments our learning-curve. This learning-curve included 22 women, since three women were treated twice within these first 25 treatments (Table 2). Women treated during the learning-curve were significantly older (*p* = 0.005), and the NPV% immediately post-treatment was significantly lower (44.6% range: 0–99.7 versus 74.7% range 0–120.6; *p* = 0.011). The percentage of women with a reintervention after the first 25 treatments was 45.5%, compared to 18.8% after the remaining treatments (Table 3). The degree of symptom reduction and QoL improvement after the first 25 and the subsequent treatments also differed, albeit not statistical significantly (Table 4).

## Discussion

In this paper we defined our learning-curve and described the implementation process of MR-HIFU treatment of uterine fibroids in our non-academic teaching hospital. Overall, we observed a significant symptom reduction and increased QoL at three, six and twelve month’s of follow-up and reached a median NPV of 66.5% directly after MR-HIFU treatment. It became clear that most treatment failures occurred during the first 25 treatments, resulting in both increase in NPV% and decrease in reintervention rate when we compared the first 25 treatments to the remaining 51 treatments. We therefore considered the first 25 treatments our learning-curve. During implementation of MR-HIFU and evaluation of our clinical results, we identified various hurdles that needed to be overcome and lessons that needed to be learned. We ordered those lessons on the level of technique, patient selection, patient counseling, medical specialists and organization (Table 6) and comment on most of these in this section.

**Table 6:** Different identified barriers and lessons learned on technical, patient selection, patient counseling, medical specialist and organizational level

Level	Barriers	Lessons learned
Technique	1. Malfunction of device 2. Treatment failures resulting in low NPV% - Bowel/ovaries in sonication beam pathway - Abdominal scar in sonication beam pathway - Abortion of sonication as a result of experienced pain - Motion artifacts in case of small fibroids	1. Ensure well-trained technical medical staff 2. Facilitate site visitation by proctor before start 3. Train team after every update of device 4. Ensure the possibility of remote consultation of device manufacturer 5. Optimize manipulation protocol 6. Ensure continuous feedback from patient during treatment 7. Be able to perform alterations in treatment strategy: longer intermissions between sonications, wider distribution of sonication, altered wattage of sonication 8. Use a light or moderate sedation protocol with the possibility to perform patient specific alterations 9. Use breath holding instructions in case of small (< 3 cm diameter) fibroids
Patient selection	1. Low eligibility number 2. Heating failures resulting in no or low NPV% 3. High number of adverse events 4. Misinterpretation of retention bladder 5. Low NPV% resulting in high reintervention rate 6. No uniformity in collected MRI data leading to difficulties in assessing eligibility 7. No uniformity in collected MRI data of treatment effect in follow-up	1. Expend inclusion criteria based on recent literature and gained experience 2. Keep in mind that multiple inclusion criteria combined can lead to unsuitable patients 3. Use the latest equipment version including an integrated cooling system 4. Keep in mind that a uterine fibroid on a bladder ultrasound, performed after removal of a catheter, can be mistaken for urinary retention and therefore lead to unnecessary interventions 5. Manipulation and sedation protocol optimization can contribute to a high NPV% 6. Development of MRI scan review templates, either for screening, treatment or follow-up, leads to uniform data collection
Patient counseling	1. Inadequate counseling 2. To high expectations of treatment effect	1. Facilitate additional counseling performed by a direct involved member of the treatment team 2. Emphasize on realistic expectations of MR-HIFU treatment and timespan of treatment effect
Medical specialists	1. Fear for loss of income at gynecology department 2. Responsibility for patient on treatment day	1. Collect referral data 2. Perform substitution analysis 3. Appoint a medical specialist who is responsible during screening, on the treatment day and during follow-up
Organization	1. Unfamiliar with implementation of new treatment option 2. Lack of research department in non-academic hospital 3. Lack of nursing ward in radiology department and unfamiliarity with MR-HIFU treatment on nursing ward 4. Sparse MRI scanner time and time-consuming preparations	1. Invest in infrastructure (e.g., a research unit) to smoothen the implementation process 2. Involve all responsible parties (e.g., medical specialists) from the start to feel jointly responsible for success of implementation 3. Train nurses and develop a standardized nursing protocol 4. Develop a Standardized Operating Procedure (SOP) besides a nursing protocol to save sparse MRI scanner time and improve both efficiency and safety 5. Add administration of a uterus stimulant during treatment to improve sonication efficiency

### Technical level

On top of the 76 described treatments in the result section, three additional treatments were planned, but treatment could not take place. Twice this was due to malfunction of the device and once due to a power cut at the radiology department. No solution could be found during the treatment, even after consulting the technical team of our hospital and the experts of the vendor. During two other treatments, that were included in the result section, malfunction of the device occurred as well, leading to a delay of treatment and/or the decision to stop the treatment prematurely because of lack of time. We therefore believe that manufacturers should continue to focus on prevention of these malfunctions and these problems emphasize the importance of well-trained technical staff that can be consulted when needed.

The advantages we experienced by using a manipulation protocol, and the advantages of the protocol we used, were described before [5, 20, 21] and included the following three steps: (1) the BRB (bladder filling, rectal filling, bladder emptying) maneuver with adjusted rectal filling by adding psyllium fibers to the solution; (2) trendelenburg position combined with bowel massage; (3) the manual uterine manipulation (MUM) method for uterine repositioning. Verpalen et al. showed the eligibility improvement of our patient population after implementing this manipulation protocol in detail before [21].

Women with an abdominal scar in the beam pathway could be treated by repositioning the patient to avoid skin burns and without using a scar pad [8]. As described before by Mindjuk et al., in case heating through the scar is unavoidable, special attention to near field heating close to the skin, combined with the patients' feedback experiencing pain, is required and results in more safe treatments. Furthermore, longer intermissions between sonications, wider distribution of the sonications in the fibroid or use of a lower wattage are advised [5].

To reduce failures as a result of experienced pain by the patient, our sedation protocol was optimized. Procedural Sedation and Analgesia (PSA) is increasingly used during uncomfortable radiological interventions and is also suitable for the MR-HIFU treatment of fibroids [22, 23]. Sedation is performed to prevent the patient from deep visceral pain, hot sensations on the skin and motion artifacts. Light to moderate sedation results in regular breathing patterns and quick recovery, whereas deep sedation can lead to irregular breathing patterns and involuntary motions. These instable breathing patterns and involuntary motions can complicate the procedure and communication of patients about pain or discomfort during the procedure, which could lead to adverse events like skin burns [23]. Initially we used only light sedation, but after 25 patients we liberated our protocol and left more room for an increased administration of both sedatives and analgesics to a moderate sedation level (Additional file 1).

The six remaining failures during treatment, in which the fibroid could not be adequately heated, occurred when we extended our inclusion criteria. In retrospect, we had become overconfident after being able to manage the previous described treatment failures, and started including fibroid types not suitable for MR-HIFU treatment.

When analyzing all failures in time, it became clear that most treatment failures occurred within the first 25 treatments and therefore we considered this our learning-curve. Earlier studies reported the existence of a learning-curve during implementation of the MR-HIFU treatment of uterine fibroids [24, 25]. Okada et al. observed a significant increase in NPV% and decrease in reinterventions when comparing the first 144 treatments performed in four different clinics (not equally distributed) to the second 143 performed treatments [11]. Mindjuk et al. also mentioned an increase in NPV% due to learning-curve effect and in a previous publication of the same group, the improvement of technique was appointed to be a main reason for this clinical treatment improvement [5, 26]. This is in line with our study showing that the NPV% achieved immediately post-treatment increased significantly after the first 25 treatments from 44.6 to 74.7%. An NPV of 74.7% is similar to other studies using a full-ablation protocol, reporting NPV percentages between 45.4 and 97.7%. Furthermore, our  mean decrease in fibroid volume of 42.4% at 6-month follow-up, is comparable with previous literature as well, with a  median fibroid volume decrease of 36.6% after 6 months in the systematic review by Verpalen et al. [16]. After overcoming our learning-curve, we had a reintervention rate of 18.8%, with a median follow-up of 24 months, which is comparable to UAE (20% in Volkers et al.), but higher compared to the previous publication by Mindjuk et al. (12.7%, mean NPV 88.7%) with a comparable mean follow-up of 19.4 months [5, 27]. This is most likely the result of our lower NPV%. As Mindjuk et al. emphasized in their paper, reintervention rate is closely related to NPV% and an NPV% above 80% leads to clinical success rate in 81%, compared to 51% in case of an NPV below 80% [5].

### Patient selection

When our initial inclusion and exclusion criteria were applied to the women participating in our MaSSI study, 47.6% (80/168) of women would have been eligible for MR-HIFU treatment. We found that the risk on failure was particularly high in case of deep sonications (10–12 cm from skin to fibroid) and/or a thick abdominal fat layer (3–4 cm) in combination with high signal intensity on the T2-weighted MRI scan (Funaki 2 or 3). When this combination of factors is present, restraints should be exercised in the decision to treat this patient.

As we gained experience, we adjusted our inclusion and exclusion criteria to increase eligibility, particularly fibroids classified as Funaki 3 fibroids. We experienced that several Funaki 3 fibroids could successfully be treated, although including high signal intensity fibroids also led to treatment failures. Therefore at this point, we are reluctant to include Funaki 3 fibroids.

Initially, we intended to treat only women with one fibroid. However, if more than one but less than five fibroids seemed to cause symptoms, more fibroids were treated from June 2017 (after 22 treatments) onward. In October 2017 (after 26 treatments) we implemented our new manipulation protocol [21], and from November 2017 (after 29 treatments) onward, future pregnancy wish was no reason for exclusion anymore. These changes also led to increased eligibility of patients. After extending our inclusion criteria, we retrospectively analyzed that our eligibility rate would have been 69.6% (117/168) when applied on all MaSSI participants. This percentage is much higher than reported in other, older, studies where the eligibility ranged between 23 and 27% [17, 28].

In 26% (18/70) of patients, an adverse event on treatment day occurred (Table 3) and in 4.3% (3/70) an event needed additional treatment. The use of a dated version of the device without an integrated cooling system might have caused a higher risk for health-related adverse events (1.4% of all complications) [16, 29]. In the latest version of the Sonalleve (V2 tabletop), an integrated cooling system cools down the skin temperature after every sonication. Use of the V2 might have prevented skin burns in our case.

### Patient counseling

Counseling of patients about the different fibroid treatment options, including MR-HIFU, was performed by the gynecologist. However, since MR-HIFU is performed by radiologists, additional counseling by radiologists is recommended for those patients who opt for MR-HIFU. At the beginning of our study, we experienced very high expectations of the effect of MR-HIFU, particularly concerning the time women could expect improvement. Later, more emphasis was put on realistic expectations and the timeline.

### Differences in perspective from the involved medical specialists

In order to successfully implement MR-HIFU treatment of uterine fibroids, collaboration between radiologists and gynecologists is essential [8]. Since MR-HIFU is performed at the radiology department, the gynecology department initially feared loss of revenues due to a decrease in fibroid-related surgeries after MR-HIFU implementation. However, the implementation of the MR-HIFU treatment led to a higher referral rate from other institutions so that the total number of patients in need for surgical treatment options did not decrease. In order to manage expectations of all stakeholder, we advise to register patients’ referral patterns and costs of alterations in these patterns for both radiology and gynecology departments. This registration can be used as input for a budget impact substitution analysis to predict potential negative financial consequences for both the gynecology and radiology department by loss of revenues and increased costs, respectively.

Since the MR-HIFU treatment was performed by the radiologist and the follow-up was handled by the gynecologist, clear agreements needed to be made on who had which responsibilities at what stage with regard to the patient. We decided in our institution that the radiologist was responsible during treatment and the following 24 h, the gynecologist was responsible from 24 h after treatment onward.

### Organization level

Implementation of a new treatment can be challenging, and publications on process evaluation or implementation strategy are scarce in general [30]. We appointed a full-time PhD candidate to support the MR-HIFU team with implementation, setting up the workflow and clinical protocols. For successfully introducing the MR-HIFU technology, we acknowledged that a multidisciplinary treatment concerning different medical specialties, requires close collaboration between departments to be successful. We therefore updated all stakeholders during the entire implementation process, which is highly recommended to ensure shared responsibility to make implementation successful.

Before we started with the implementation of MR-HIFU, we could not find formats such as template reports of screening MRI scans, multidisciplinary meetings and Standard Operating Procedure (SOP’s). We expected that these documents would reduce logistic barriers, would improve efficiency and effectiveness and would facilitate implementation. Since the radiology department did not have a nursing ward, nurses needed to be trained to take care of our MR-HIFU patients at the gynecology department. A standardized nursing protocol was implemented in June 2017. Additionally, we implemented preparations at the gynecology ward, such as the blather catheter, pre-medication and IV-line to increase efficiency and save valuable MRI time. All these procedures were described in an SOP that included counseling, screening, treatment and follow-up to improve the efficiency of all different stages of the MR-HIFU treatment (Additional file 1). All MR-HIFU radiologists reviewed screening MRI scans for eligibility. The development of a template MRI report for the screening MRI scan helped them collecting all the data needed to assess eligibility (Additional file 1). Similar templates were designed to ensure that uniform reports were prepared of the MRI scan immediately after MR-HIFU treatment and at 6-month follow-up.

Furthermore to improve treatment efficiency, from January 2018 we implemented the administration of a uterus stimulant in our treatment protocol at the start of sonications when no contra-indications were known (Table 6). Previous studies indicate that the use of a uterus stimulant has a beneficial effect on treatment effectivity, but its (cost)effectivity needs to be proven in future studies [31, 32].

The last remaining hurdle to take is at a societal level. Due to the lack of randomized controlled trials in which the long-term follow-up outcomes of the MR-HIFU treatment are compared with standard care, the MR-HIFU treatment is not included in Dutch national guidelines and there is no reimbursement by the health insurance companies. We strongly recommend close collaboration with the most important stakeholders (e.g., the national societies of obstetrics and gynecology, insurance companies and the hospital board) from the start of implementation of this new technique, in order to facilitate dissemination and further adoption after proven (cost)effectiveness [33].

### Strengths and limitations

MR-HIFU itself and the implementation of this multidisciplinary uterine fibroid treatment are complex, especially with the current lack of standard guidelines, and this might discourage new sites to start offering this non-invasive treatment option. In this article we reported all lessons learned, while we implemented the MR-HIFU treatment of uterine fibroids in our hospital and we provided straightforward ready-to-use protocols on how to perform sedation, suggestions for MRI examination and SOP’s on logistics in our supplements. On different levels of implementation, Table 6 can be used as an inspiration for possible hurdles that need to be overcome, although these can be rather site specific and are not inexhaustible. We believe the most important strength of this article is that by doing so, we provide other centers an overview of what is necessary to start implementing MR-HIFU for uterine fibroid treatment. Furthermore, we identified a learning-curve of 25 treatments and we believe this information is helpful for the expectation management of all involved parties of when to expect successful treatment. Finally, we addressed frequent types of MR-HIFU treatment failures and reported possible solutions that will result in higher eligibility rates and might even shorten the learning-curve.

The primary goal of our MaSS study, and this article, was not to evaluate all clinical outcomes in detail. Therefore, multiple limitations can be reported concerning the clinical outcome data collection. A high lost-to-follow-up number was seen, partly due to reinterventions, which might have led to an overestimation of clinical symptom and QoL improvement, although outcomes are in line with current literature. Baseline characteristics of our first 25 patients differed from the remaining patients, probably leading to favorable and unfavorable situations, and the follow-up duration varies between the first 25 and the subsequent group, although follow-up was at least one year and most reinterventions of the learning-curve took place within the first year. Moreover, the use of oral contraceptives or intra-uterine devices after MR-HIFU treatment could have interfered with symptom improvement. Nevertheless, we believe this does not curtail the usefulness of our lessons learned.

Since the improvement of our counseling, screening and treatment protocols took place continuously during inclusion , identifying which of them contributed to the change in clinical outcome is challenging. The cutoff point used for the analysis of our clinical outcomes was somewhat arbitrary. However, despite we broadened eligibility and included more complicated cases, after completing our learning-curve, failure rate decreased and relevant outcomes like NPV% and reintervention rate improved.

We used an adjusted process evaluation model when retrospectively evaluating our implementation process. For future analyses we recommend to prospectively evaluate the implementation processes, since this ease the process and can be used for quality improvements [34].

### Future perspectives

Some hurdles still have to be overcome in order to reach complete adoption of the MR-HIFU treatment of uterine fibroids. When it comes to clinical outcomes, improvement can be reached by further optimizing screening. On a technical level, tools to sonicate fibroids with high signal intensity and techniques to measure NPV% during treatment are necessary to further increase eligibility and shorten treatment time. Randomized controlled trials comparing long-term (cost)effectiveness of MR-HIFU with standard fibroid care, from both clinical and societal perspective, are needed.

## Conclusion

In this article we identified our learning-curve by analyzing our clinical results, and we presented the implementation of uterine fibroid MR-HIFU treatment in our non-academic teaching hospital. Our lessons learned on a technical, patient selection, patient counseling, medical specialists and organizational level, are described in detail, and the provided supplements are likely to be of benefit to other hospitals willing to commence with offering MR-HIFU as novel treatment option to women with symptomatic uterine fibroids.

## Supplementary Information

**Additional file 1.** Supplement 1: MRI protocol. Supplement 2: Standard Operating Procedure (SOP). Supplement 3: Sedation protocol.

## Data Availability

The datasets used and/or analyzed during the current study are available from the corresponding author on reasonable request.

## Abbreviations

BRB: Bladder filling, Rectal filling, Bladder emptying maneuver
ISP: IntelliSpace Portal
MaSS: Myoma Screening Study
MRI: Magnetic Resonance Imaging
MR-HIFU: Magnetic Resonance image-guided High-Intensity Focused Ultrasound
MUM: Manual Uterine Manipulation
NPV: Non-Perfused Volume
PSA: Procedural Sedation and Analgesia
SOP: Standard Operating Procedure
tHRQL: transformed Health-Related Quality of Life score
tSSS: transformed Symptom Severity Score
UFS-QoL: Uterine Fibroid Symptom and Health-Related Quality of Life questionnaire

## References

[1] Stewart EA (2001). Uterine fibroids. Lancet.

[2] Pérez-López FR, Ornat L, Ceausu H (2014). EMAS position statement: management of uterine fibroids. Maturitas.

[3] de Bruijn A, Huisman J, Hehenkamp W (2019). Implementation of uterine artery embolization for symptomatic fibroids in the Netherlands: an inventory and preference study. CVIR Endovasc.

[4] Siedek F, Yeo SY, Heijman E (2019). Magnetic Resonance-Guided High-Intensity Focused Ultrasound (MR-HIFU): overview of emerging applications (Part 2). Rofo.

[5] Mindjuk I, Trumm CG, Herzog P (2014). MRI predictors of clinical success in MR-guided focused ultrasound (MRgFUS) treatments of uterine fibroids: results from a single centre. Eur Radiol.

[6] Anneveldt KJ, van’t Oever HJ, Nijholt IM (2021). Systematic review of reproductive outcomes after high intensity focused ultrasound treatment of uterine fibroids. Eur J Radiol.

[7] Verpalen IM, Anneveldt KJ, Vos PC (2020). Use of multiparametric MRI to characterize uterine fibroid tissue types. MAGMA.

[8] Rueff LE, Raman SS (2013). Clinical and technical aspects of MR-guided high intensity focused ultrasound for treatment of symptomatic uterine fibroids. Semin Intervent Radiol.

[9] Verpalen IM, de Boer JP, Linstra M (2020). The Focused Ultrasound Myoma Outcome Study (FUMOS); a retrospective cohort study on long-term outcomes of MR-HIFU therapy. Eur Radiol.

[10] Ciebiera M, Łoziński T (2020). The role of magnetic resonance-guided focused ultrasound in fertility-sparing treatment of uterine fibroids-current perspectives. Ecancermedicalscience.

[11] Okada A, Morita Y, Fukunishi H (2009). Non-invasive magnetic resonance-guided focused ultrasound treatment of uterine fibroids in a large Japanese population: impact of the learning curve on patient outcome. Ultrasound Obstet Gynecol.

[12] Funaki K, Fukunishi H, Funaki T (2007). Magnetic resonance-guided focused ultrasound surgery for uterine fibroids: relationship between the therapeutic effects and signal intensity of preexisting T2-weighted magnetic resonance images. Am J Obstet Gynecol.

[13] Spies JB, Bradley LD, Guido R (2010). Outcomes from leiomyoma therapies: comparison with normal controls. Obstet Gynecol.

[14] Fennessy FM, Tempany CM, McDannold NJ (2007). Uterine leiomyomas: MR imaging–guided focused ultrasound surgery—results of different treatment protocols1. Radiology.

[15] Hesley GK, Gorny KR, Henrichsen TL (2008). A clinical review of focused ultrasound ablation with magnetic resonance guidance: an option for treating uterine fibroids. Ultrasound Q.

[16] Verpalen IM, Anneveldt KJ, Nijholt IM (2019). Magnetic resonance-high intensity focused ultrasound (MR-HIFU) therapy of symptomatic uterine fibroids with unrestrictive treatment protocols: a systematic review and meta-analysis. Eur J Radiol.

[17] Ikink M, Voogt M, Verkooijen H (2013). Mid-term clinical efficacy of a volumetric magnetic resonance-guided high-intensity focused ultrasound technique for treatment of symptomatic uterine fibroids. Eur Radiol.

[18] Dindo D, Demartines N, Clavien PA (2004). Classification of surgical complications: a new proposal with evaluation in a cohort of 6336 patients and results of a survey. Ann Surg.

[19] Wensing M, Grol R (2017). Implementatie Effectieve verbetering van de patiëntenzorg.

[20] Kim YS, Bae DS, Park MJ (2014). Techniques to expand patient selection for MRI-guided high-intensity focused ultrasound ablation of uterine fibroids. AJR Am J Roentgenol.

[21] Verpalen IM, van’t Veer-Ten Kate M, de Boer E (2020). Development and clinical evaluation of a 3-step modified manipulation protocol for MRI-guided high-intensity focused ultrasound of uterine fibroids. Eur Radiol.

[22] Yao C-L, Trinh T, Wong GTC (2008). Anaesthesia for high intensity focused ultrasound (HIFU) therapy. Anaesthesia.

[23] Vaessen HHB, Knuttel FM, van Breugel JMM (2017). Moderate-to-deep sedation technique, using propofol and ketamine, allowing synchronised breathing for magnetic resonance high-intensity focused ultrasound (MR-HIFU) treatment for uterine fibroids: a pilot study. J Ther Ultrasound.

[24] Ikink ME, Nijenhuis RJ, Verkooijen HM (2014). Volumetric MR-guided high-intensity focused ultrasound versus uterine artery embolisation for treatment of symptomatic uterine fibroids: comparison of symptom improvement and reintervention rates. Eur Radiol.

[25] Thiburce AC, Frulio N, Hocquelet A (2015). Magnetic resonance-guided high-intensity focused ultrasound for uterine fibroids: Mid-term outcomes of 36 patients treated with the Sonalleve system. Int J Hyperth.

[26] Trumm CG, Stahl R, Clevert D (2013). Magnetic resonance imaging-guided focused ultrasound treatment of symptomatic uterine fibroids impact of technology advancement on ablation volumes in 115 patients. Invest Radiol.

[27] Volkers NA, Hehenkamp WJK, Birnie E, Ankum WM, Reekers JA (2007). Uterine artery embolization versus hysterectomy in the treatment of symptomatic uterine fibroids: 2 years’ outcome from the randomized EMMY trial. Am J Obstet Gynecol.

[28] Mohr-Sasson A, Machtinger R, Mashiach R (2018). Long-term outcome of MR-guided focused ultrasound treatment and laparoscopic myomectomy for symptomatic uterine fibroid tumors. Am J Obstet Gynecol.

[29] Browne JE, Gorny KR, Hangiandreou NJ (2020). Comparison of clinical performance between two generations of magnetic resonance-guided focused ultrasound systems in treatments of uterine leiomyomas. Acad Radiol.

[30] Craig P, Dieppe P, Macintyre S (2008). Developing and evaluating complex interventions: the new Medical Research Council guidance. BMJ.

[31] Lozinski T, Filipowska J, Krol P (2018). Oxytocin administration in high-intensity focused ultrasound treatment of myomata. Biomed Res Int.

[32] Jeong JH, Hong GP, Kim YR (2016). Clinical Consideration of treatment to ablate uterine fibroids with Magnetic Resonance Imaging-guided High Intensity Focused Ultrasound (MRgFUS): Sonalleve. J Menopausal Med.

[33] Leviton LC, Melichar L (2016). Balancing stakeholder needs in the evaluation of healthcare quality improvement. BMJ Qual Saf.

[34] Hulscher MEJL, Laurant MGH, Grol RPTM (2003). Process evaluation on quality improvement interventions. Qual Saf Health Care.

