# Supplementary material for: Lessons learned during implementation of MR-guided High-Intensity Focused Ultrasound treatment of uterine fibroids
Source: Insights Imaging. 2021 Dec 18;12:188. doi: 10.1186/s13244-021-01128-w (PMC8684568; doi:10.1186/s13244-021-01128-w)
Supplement: Supplementary file 1 — Additional file 1. Supplement 1: MRI protocol. Supplement 2: Standard Operating Procedure (SOP). Supplement 3: Sedation protocol. [file 13244_2021_1128_MOESM1_ESM.pdf]

Additional file 1

Supplement 1: MRI protocol

Screening MRI protocol

	**T2w axial**	**T2w sagittal**	**T2-mapping**	**DWI**	**DWI long TE**	**CE-T1w**
Scan type	TSE multishot<br>TSE factor 18	TSE multishot<br>TSE factor 17	GRASE multishot<br>TSE factor 12<br>EPI factor 5	Single shot<br>SE-EPI<br>EPI factor 51	Single shot<br>SE-EPI<br>EPI factor 51	3D FFE<br>TFE multishot<br>TFE factor 44
T_E (ms)	110	125	n x 20	64	140	2.6
T_R (ms)	3656	6219	2438	2673	6715	5.4
Flip angle (˚)	90	90	90	90	90	10
Slice thickness (mm)	3.5	3.0	7.0	7.0	7.0	3.0
ACQ Matrix	356x198	356x187	112x82	112x80	112x80	168x157
FOV (mm)	250x180	250x180	250x188	250x188	250x188	250x250
ACQ Voxel size (mm)	0.7x0.88	0.7x0.94	2.23x2.23	2.23x2.26	2.23x2.26	1.5x1.49
Scan%	77.3	73.0	98.2	95.6	95.6	94
NSA	2	3	2	4	4	3
Half scan	No	No	No	Yes	Yes	No
Fat supression	No	No	SPIR	SPAIR	SPAIR	SPAIR
Scan duration (min:s)	2:48.2	3:31.5	2:55.5	6:51.6	4:35.3	2:31.2

DWI: Diffusion Weighted Imaging; T_E: echo time; TSE: Turbo Spin Echo; GRASE: gradient and spin echo; EPI: Echo Planar Imaging; SE: Spin Echo; FFE: Fast Field Echo; TFE: Turbo Field Echo; T_R: repetition time; ACQ: acquired; FOV: Field of View; Scan%: Scan percentage; NSA: Number of Signal Averages; SPIR: spectral presaturation inversion recovery; SPAIR; Spectral Selection Attenuated Inversion Recovery

Screening MRI report

Indication
Symptomatic uterus myomatosus, eligible for MR-HIFU treatment?

Medical history
…

Report
MRI female genitals including contrast
Used sequences: T2-weighted, T1-weighted after contrast, T2 mapping (GRASE) and diffusion weighted MRI. No vaginal or rectal contrast infusion.

Position uterus: anteflexion/interposition/retroflexion
Bowel interposition: yes/no
Subcutaneous fat layer: …cm

Fibroid
Type: submucosal/intramural/subserosal and FIGO classification (0-7)
Location: anterior/posterior/in fundo and/or left/right dorsal/ventral located
Diameter of fibroid: …cm
Distance center fibroid to sacral plexus: …cm
Distance fibroid to subcutaneous fat layer: …cm
Funaki classification: 1/2/3
Contrast enhancement: homogeneous/heterogeneous/no enhancement

Other fibroids
…

Incidental findings
…

Conclusion
...

MR-HIFU treatment report

Indication
Symptomatic uterus myomatosus

Report of treatment
Counseling performed and recorded in patient file
Informed consent collected and stored in patient file

Pre medication provided: diclofenac 100mg, paracetamol 1000mg and oxycodone 10mg
Preparation on nursing ward without irregularities: enema, catheter, intravenous line.

Time of arrival at MRI: …h
Time Out Procedure performed.

Start of positioning: …h
Scout images show: uterus in anteflexion/interposition/retroflexion.
Interposition of bowels: yes/no
Manipulation provided: yes/no
Type of manipulation: BRB with/without metamucil and/or bowel massage and/or uterus manipulation

Start of treatment: …h
An MR-HIFU treatment is performed.
The treatment was successful: yes/no
Heating of fibroid was with/without notifications
Number of sonications: …
Particularities: …

End of treatment: …h
After contrast administration: …% NPV and …cm^3
Particularities: …
The % NPV was expected: yes/no
MRI room available for next patient: …h

Conclusion
MR-HIFU treatment with bad/moderate/good technical result and a NPV of …%

Six months follow-up MRI report

<u>Medical indication</u>
Symptomatic uterus myomatosus

<u>Research question</u>
Six month follow-up MRI scan after MR-HIFU treatment

<u>Report</u>
MRI female genitals including contrast
Used sequences: T2-weighted, T1-weighted after contrast, T2 mapping (GRASE) and diffusion weighted MRI. No vaginal or rectal contrast infusion.

<u>NPV</u>
NPV post MR-HIFU treatment was …cm^3 and is …cm^3 at this point.
Further decrease is expected: yes/no.

<u>Dimensions treated fibroid</u>
Fibroid measured …x… cm and is …x…cm at this point. Therefore, decrease/increase of the fibroid is measured.

<u>Other fibroids</u>
…

<u>Incidental findings</u>
…

<u>Conclusion</u>
Volume reduction of the treated fibroid is achieved: yes/no
More decrease in size is expected: yes/no.

Supplement 2: Standard Operating Procedure (SOP)

Preparation

**Action**	**Person in charge**
Diagnosing patient with uterus myomatosus and screening for MR-HIFU treatment eligibility	Gynecologist
Counseling patient. Signing informed consent	Gynecologist
Ordering screening MRI scan	Gynecologist
Reviewing screening MRI scan and ordering multidisciplinary meeting	Radiologist
Attending multidisciplinary meeting	Gynecologist<br>Radiologist<br>PhD candidate
Recording results of multidisciplinary meeting in patient file	Gynecologist
Giving feedback of meeting to patient, ordering MR-HIFU treatment	Gynecologist
Planning MR-HIFU treatment including appointment at the anesthesia department and short stay	Secretary
Counseling of patient regarding sedation protocol including use of uterus stimulant during treatment	Anesthetist
Preparing patient for hospital admission	Secretary

Pre-procedural

**Action**	**Person in charge**
Prescribing pre-medication on day of treatment	Radiologist
Preparing patients as described in nursing protocol	Nurse
Performing time-out procedure before start treatment at MRI scanner	Radiologist
Positioning of patient on MRI scanner	MRI technicians

Peri-procedural

**Action**	**Person in charge**
Performing MR-HIFU treatment	Radiologist

Post-procedural

**Action**	**Person in charge**

Inspection of abdominal skin at MRI scanner	Radiologist
Performing rounds after treatment at general nursing ward	Radiologist
Documenting treatment in patient file, sending discharge letter to general practitioner	Radiologist
Ordering follow-up appointments	Radiologist

Supplement 3: Sedation protocol

Preparation

All patients are screened for eligibility for Procedural Sedation and Analgesia (PSA) and use of a uterus stimulant during a consultation at the anesthesiology department. Patients need to arrive sober on treatment day (for at least 6 hours). At the general nursing ward, oral pre-medication will be administered. This includes 100mg diclofenac, 1000mg paracetamol and 10mg oxycodone. At the nursing ward, an intravenous cannula will be applied for continuous infusion of 0.9% saline.

Necessities at the MRI scanner

Sedation trolley including MRI compatible CO_2 and O_2 monitoring materials, suction materials, perfusion materials, cage of Faraday and sedation (emergency) medication including:

- atropine
- ephedrine
- phenylephrine
- fentanyl
- granisetron
- propofol
- NaCl 0.9%
- carbetocin 100µg/ml

Work plan during MR-HIFU treatment

A sedation professional continuously monitors patients' vital signs with a three-lead electrocardiogram (ECG), pulse oximetry (SpO2), non-invasive blood pressure (NIBP) measurement, measured at 5-minute intervals or more frequently when needed, and continuous capnography. All patients receive supplemental oxygen (2 L/min) by nasal cannula.

Propofol, 20mg/mL is administered by a continuous infusion pump on a rate between 1mL/hour and 12mL/hour. In case necessary, propofol can be increased with 0.5ml/h or 1ml/h or a bolus of 10mg/1mL. When pre-medication effects decreases, fentanyl bolus can be administered (25µg/0.5mL or 50µg/1mL). During treatment the sedation professional communicates with the patient on a regular basis between sonications.

After first successful sonication, one ampoule of 100µg/ml carbetocin is slowly intravenously administered.

Recovery

When treatment is finished, recovery will take place on the general nursing ward. Patient' vital signs will be measured according nursing protocol and more frequent when necessary.
